# Gazing into the flames: A guide to assessing the impacts of climate change on landscape fire

**DOI:** 10.1126/sciadv.adz2429

**Published:** 2025-12-19

**Authors:** Hamish Clarke, Francesca Di Giuseppe, Lynn Johnston, Jennifer Marlon, Trent Penman, Andrew J. Pitman, Guido R. van der Werf, Mike D. Flannigan

**Affiliations:** ^1^ FLARE Wildfire Research, School of Agriculture, Food and Ecosystem Sciences, University of Melbourne, Parkville, Victoria, Australia.; ^2^ European Centre for Medium-range Weather Forecast (ECMWF), Reading, UK.; ^3^ Natural Resources Canada, Canadian Forest Service, Sault Ste. Marie, Ontario, Canada.; ^4^ School of the Environment, Yale University, New Haven, CT, USA.; ^5^ ARC Centre of Excellence for Climate Extremes and Climate Change Research Centre, University of New South Wales, Sydney, Australia.; ^6^ Environmental Sciences Group, Wageningen University & Research, Wageningen, Netherlands.; ^7^ Natural Resource Science, Thompson Rivers University, Kamloops, British Columbia, Canada.

## Abstract

Widespread impacts of landscape fire on ecosystems, societies, and the climate system itself have heightened the need to understand the potential future trajectory of fire under continued climate change. However, the complexity of fire makes climate change impact assessment challenging. The climate system influences fire in many ways, including through vegetation, fuel dryness, fire weather, and ignition. Furthermore, fire’s impacts are highly diverse, spanning threats to human and ecological values and beneficial ecosystem and cultural services. Here, we discuss the art and science of projecting climate change impacts on landscape fire. This not only includes how fire, its drivers, and its impacts are modeled, but critically it also includes how projections of the climate system are developed. By raising and discussing these issues, we aim to foster the development of more robust and useful fire projections, help interpret existing assessments, and support society in charting a course toward a sustainable fire future.

## Teaser

An understanding of fire and climate modeling must inform assessments of climate change impacts on landscape fire.

## INTRODUCTION

Less than 200 years of human activity—principally via the burning of fossil fuels and clearing of land—has led to changes that register as anomalous in the geological record (*1*). This is a remarkable pace of change, even when considering our planet’s climate system is intrinsically dynamic and has oscillated between warmer and cooler periods since Earth’s formation well over 4 billion years ago (*2*). These changes include planetary surface air temperatures higher than any multicentury period in the past 100,000 years, atmospheric methane and nitrous oxide concentrations at their highest levels in at least 800,000 years, and carbon dioxide levels that are unprecedented for at least 2 million years (*1*). For the past 10% of its 4.5–billion year history, Earth’s climate system—and much of its biodiversity—has evolved alongside the dynamic and fluctuating presence of landscape fire (*3*). The advent of global fire monitoring has revealed new detail about the spatiotemporal variability of fire over recent decades, including declining trends in area burnt in savannahs and increases in extratropical forests (*4*, *5*). A picture is emerging of rapid changes in the drivers and impacts of fire, but many uncertainties remain given the inherent complexity of landscape fire and the relatively short observational record (*6*).

Landscape fire is a prototypical extreme-driven system. For many vegetation types, a small number of large fires account for most of the area burned (*7*). Similarly, for the human, social, economic, and environmental impacts of landscape fire, infrequent but unusually large fire events and seasons tend to dominate impacts. For example, about 15 million hectares burnt during the 2023 fire season in Canada, more than double the area of the previous biggest season, generating record carbon and smoke emissions and more than 80% of the pyrocumulonimbus events observed on earth that year (*8*). The Black Summer fires of 2019 to 2020 in eastern Australia set global records for annual proportion of forest biome burnt (*9*), exposing ecosystems to an unprecedented areal extent of high-severity landscape fire (*10*). In recent years, increases have been observed in the frequency and intensity of extreme fire weather conditions (*11*) and in the high-intensity, high-impact fire events they generate (*12*). Given this centrality of extremes in driving fire activity and impacts, there is a critical need to understand the conditions that foster such events (*13*).

**Fig. 1.**
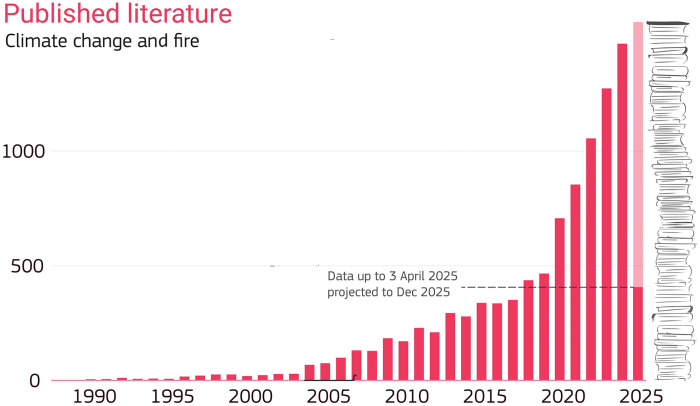
The trend in the number of journal articles published on the topic of climate change and landscape fire. See the Supplementary Materials for details of search term.

Similar to the current changes in the climate system itself, recently observed shifts in fire activity are expected to be followed by much greater changes in the future in the absence of rapid and deep emission reductions (*14*). There is an urgent need to understand and plan for the fires of the future. So how do we do that? For strongly weather and climate-driven hazards such as heatwaves, drought, flooding, and tropical cyclones, there is a well-established (although still complex) path toward estimating the projected future impacts of climate change. This involves using Earth system models (ESMs), mathematical models that represent the physical laws that govern the earth system (*15*). By specifying projected changes in anthropogenic greenhouse gasses, solar irradiance, volcanic activity, and other climate forcings, these models simulate the future behavior of the climate system. Notwithstanding the strong coupling between fire and climate, this standard pathway for projecting future climate is problematic for fire because its incidence and impacts are driven by a range of factors not typically included in ESMs, including the type, amount, and dryness of vegetation, ignition from lightning and human sources, the location, layout and design of human settlements, fire management activities, and other human behavior before, during, and after fires. ESMs can still be used to project future fire risk, but such projections cover only a subset of drivers (e.g., fire weather conditions) and must be coupled with other lines of evidence to arrive at a more robust assessment. Despite these limitations, there is a broad and rapidly growing literature on the future trajectory of landscape fire, using ESMs and a range of other empirical, process-based, and scenario-driven approaches [Fig. 1; see (*14*) for a review].

The aim of this paper is to review the way climate change impact assessments for landscape fire are conducted. We begin with an overview of our current understanding of landscape fire across three key domains: fire activity, the drivers of fire, and fire impacts. We then consider the fundamentals of climate change impact assessment. This section may seem like a detour to some readers due to its absence of fire-focused material, but it is precisely to support better application of this knowledge to fire that we present it as a discrete whole. This knowledge is a prerequisite to understanding how ESMs are combined with fire models to develop future fire projections, the topic of the final section. Throughout, we draw attention to issues requiring special consideration because of their complexity, uncertainty, or contentiousness. We conclude with two examples of potential research that could support a climate change impact assessment for landscape fire. In reviewing these issues, our intention is to foster the development of robust and useful fire projections to help interpret existing assessments and to support society in working toward a more sustainable fire future.

## CURRENT UNDERSTANDING OF LANDSCAPE FIRE

### Recent and historical fire patterns

**Fig. 2.**
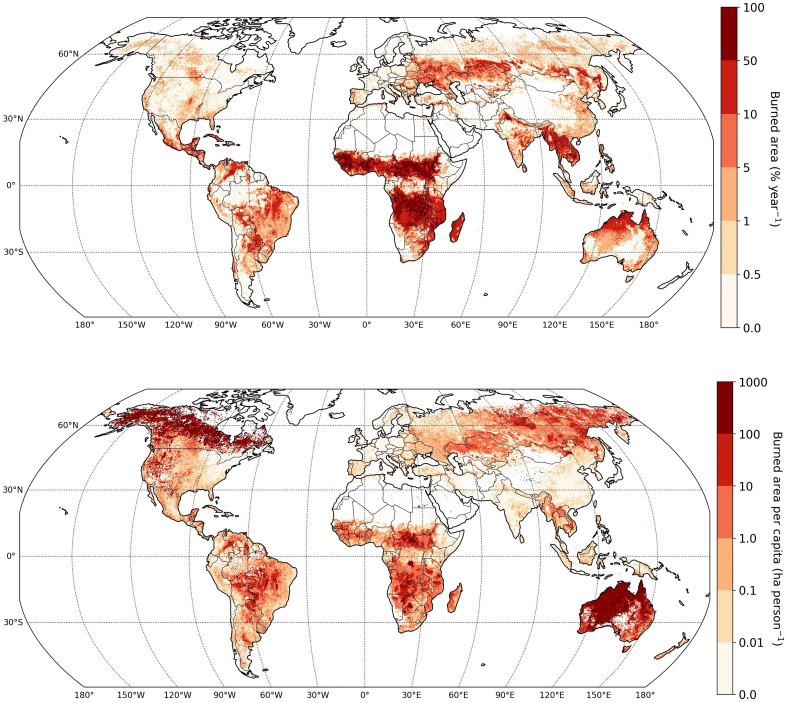
Global patterns in fire activity. Mean annual burned area (top) and mean annual burned area per capita (bottom), each expressed as a fraction of each 0.25° grid cell, averaged over 2002 to 2022. Source: (*18*, *29*).

Nowadays, most information about the location and extent of fires is derived from satellites, owing to the various sensors and algorithms detecting either area burned or active fires that have been developed since the 1990s to map fires on large scales (*16*, *17*). A first-order feature of the resulting maps (Fig. 2, top) is the dominance of the tropics and especially savanna ecosystems where wet seasons promote fuel build up and dry seasons make these fuels flammable, providing ideal conditions for fires. The sparse trees in these ecosystems have thick bark and are adapted to low-intensity fire. These low-intensity fires are integral to savanna ecosystems, which coevolved with fire, yet burned area is declining in many savannas due to increased land fragmentation and expanding agriculture (*4*). Relatively low-intensity fires are also used to clear land after harvest in areas such as the Punjab region in India, resulting in poor air quality in nearby cities that affects the health of millions of people. Of the total global burned area of around 770 Mha annually (*18*)—roughly the area of Australia—about 95% stems from fires in savanna and grasslands (85%) and agriculture (10%).

Sparsely populated fire-prone regions including western North America, boreal Canada and Siberia, and interior Australia, emerge as global hot spots when burned area is calculated per person (Fig. 2, bottom). This demographic lens highlights two interconnected dynamics. First, the world’s most extensive landscape fires occur in remote, often inaccessible areas with minimal firefighting infrastructure and limited suppression capacity. This raises questions about our capacity to respond to changes in future fire in these regions. Second, these remote fires generate consequences that transcend geography and governance, with smoke plumes traveling thousands of kilometers to degrade air quality in distant population centers, creating a mismatch between where fires ignite and where health impacts occur. Together, the per-capita map helps explain why wildfires are commanding growing public and media attention outside the tropics: Fires are critically important in most populated regions, although most global burned area lies in savannas and grasslands.

While forest fires represent a relatively small fraction of total global burned area, their dense fuel loads and vulnerable carbon stores make them disproportionately critical for Earth’s climate system. These fires fall into distinct patterns shaped by geography and human influence: Tropical forest fires are often [but not always (*19*)] intentionally ignited to clear land due to socioeconomic pressures such as demand for commodities including soy and beef, while forest fires in temperate and boreal regions are more closely linked to ecological cycles altered by humans and climate change (*5*). It is also here that some of the most devastating fire events have been recorded in recent years, including the 2019–2020 Australian Black Summer fires in the southeast of the continent, which burned 21% of the forests in that region (*9*) and the 2023 Canadian fires, which burned 5% of the Canadian forest and emitted around 650 Tg C in a single fire season (*20*). A poleward expansion of the number and extent of forest fires has been evident in the past few decades, particularly during the summers of 2019, 2020, 2023, and 2024 (*8*, *21*).

Paleofire research places modern landscape fire variations in a long-term context, providing valuable insights into how the location and prevalence of fire have shifted over millennia in response to changes in climate, vegetation, and human activity. Fire histories have been reconstructed using proxies such as fire-scarred trees, charcoal deposits in sediments and isotopes, or combustion residues in ice cores (*22*). For instance, microcharcoal abundances and morphological changes in marine sediments off the western coast of South Africa reveal cyclical variations in grassland burning driven by shifts in the intertropical convergence zone, which determines rainfall seasonality and spatial patterns. These precipitation shifts influenced grass biomass abundance and thus fuels across the continent (*23*). In Australia, Indigenous use of fire has also contributed to biocultural landscape management over tens of thousands of years, and its targeted suppression by colonial practices has directly affected landscape fire risk (*24*, *25*).

During the Holocene (past 10,700 years), the dominance of climatic influences on fire regimes gradually gave way to increasing human impacts. Early human activities altered fire ignitions, while later deforestation and land-use changes affected fuel availability. In recent centuries, fire history records from sediments, tree rings, and historical accounts reveal a recurring pattern: an initial increase in human-caused landscape fires to clear land, followed by a sharp decline, as fuels were depleted, landscapes were fragmented, and fire was actively suppressed (*26*).

This trend, as documented by the satellite record (*4*), continues today. However, it is now punctuated by the rapid emergence of severe landscape fire outbreaks, sometimes in novel locations, due to global warming, increased human-caused ignitions, and vegetation changes from human impacts (*27*). High-latitude regions, for example, are experiencing unprecedented landscape fires in areas historically frozen or covered with snow and ice. In mid-latitudes, rising temperatures in spring and fall are extending the fire season, while reduced snowpack and increased drought are drying out fuels, causing fires to grow larger and burn more intensely. Paleofire records from Alaska demonstrate marked increases in fire frequency that cannot be fully explained by human-caused ignitions, historical fire suppression, or the spread of fire-adapted invasive species (*28*). The complexity of landscape fire poses challenges for formal climate change attribution of extreme fires and fire seasons, and observed changes in fire activity, although the number of such studies is growing, particularly at a regional scale (*29*, *30*).

### Drivers of fire

**Fig. 3.**
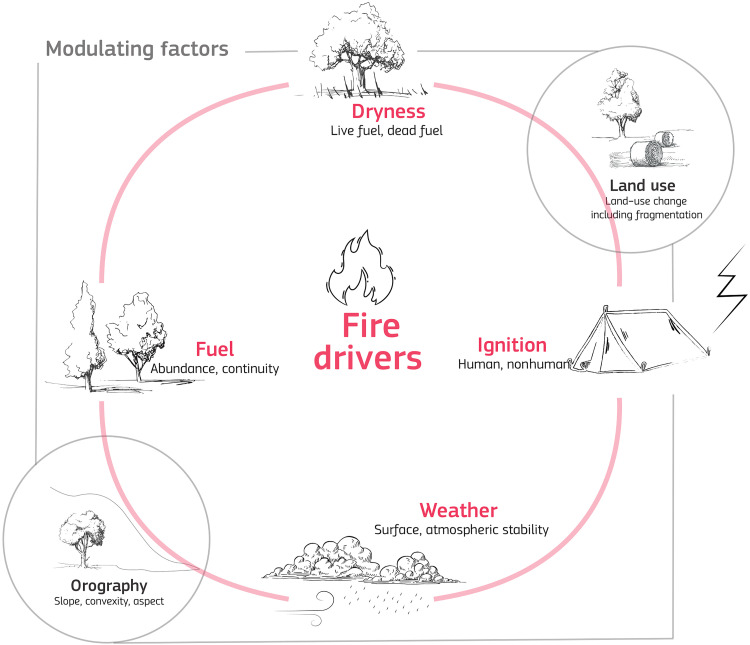
Drivers of landscape fire occurrence and propagation. Four main drivers limit the occurrence of fire: fuel amount, fuel availability (the dryness of the fuels), weather conditions (e.g., winds, temperature, and precipitation), and sources of ignition. Climate change follows multiple paths through each of these drivers to affect fire activity, often involving feedback loops.

Landscape fire initiation and spread depend on the presence and dryness of fuel, weather conditions and an ignition source [Fig. 3 and (*31*, *32*)]. These drivers are interrelated and are influenced by orography and land use. The combination of these factors at large scales defines what is known as a fire regime, encompassing a given region’s typical fire timing (e.g., frequency and seasonality), type (e.g., low-severity or crown fires), size, intensity, and severity (*33*). Weather conditions are key components of fire danger models used by fire management and emergency management agencies worldwide. These models are also used to understand how fire risk may shift under climate change (*34*). Fire danger models typically incorporate variables such as surface air temperature, relative humidity, accumulated precipitation, and near-surface wind speed to assess potential flammability and determine whether the landscape is dry enough to sustain a fire.

Fuel abundance, continuity, and dryness are critical parameters that directly influence the occurrence, intensity, and rate of spread of fires. Fuel abundance and continuity are altered by landscape fragmentation, influencing fire spread (*4*). The spatial distribution of fuel between the ground and the canopy strongly influences fire behavior and intensity (*35*). The total mass of vegetation available as fuel for burning includes both living and dead components, which differ in flammability partly due to their capacity to retain moisture.

Fuel moisture is typically expressed as the proportion of water relative to the total dry mass of vegetation and is a key determinant of flammability. Dead fuels (especially fine fuels) are the source for most fire ignitions, and they respond quickly to atmospheric conditions. In contrast, live fuels have biotic controls (e.g., stomata) that constrain moisture loss. The most severe fires often include consumption of live fuels, although this still generally requires the presence of suitable dry dead fuels (*36*). Live fuels exhibit greater spatial variability, and their moisture content depends on plant physiology, local soil conditions, and topography (*37*, *38*). Flammability of live fuels also depends on their chemical composition [in particular on volatile organic compounds (*39*)].

Topography plays a substantial role in enhancing fire spread and erratic behavior (*40*). For example, local winds generated by daytime heating create upslope and up valley winds during the day and nighttime cooling creates downslope and down valley winds (*41*). In addition, as fire moves uphill, the flames can draw closer to vegetation, preheating the fuels and increasing fire intensity. Features of slope morphology such as convexity and concavity influence the moisture content of soils and fuels, affecting their combustibility. Foehn-type winds are responsible for some extreme fire weather (*42*) and can have substantial impacts, such as the recent Los Angeles 2025 fires.

Atmospheric instability can substantially influence the spread and behavior of fires, sometimes leading to more erratic and dangerous conditions (*13*). Under unstable atmospheric conditions, fires can transition from being surface-driven, where wind dictates their behavior, to plume-driven fires. A visible indicator of plume-driven fires is the formation of a pyrocumulus cloud above the fire. If the pyrocumulus cloud grows large enough, it can begin to generate its own localized weather, including lightning, resulting in what is known as a pyrocumulonimbus—a thunderstorm created by fire (*43*). The potential for such transitions is captured in various indices and parameters derived from vertical profiles of temperature and humidity, which reflect the atmosphere’s stability.

Through the whole of human evolution, fire was used to aid survival, e.g., for hunting and managing landscapes (*44*). In many parts of the world today, ignitions are primarily associated with human activities related to pasture management, transportation, recreation, accidents, or land-use change. These activities have evolved differently according to local ecology, climate, geography, social needs, and customs. Agricultural societies in arid and semiarid biomes follow long-established indigenous practices involving low-intensity, frequent burns [e.g., Australia and South America (*45*)], while in tropical forests, fire was traditionally rare but has been introduced to accelerate land clearance for pasture and crops (*46*, *47*). In urbanized environments and at the wildland urban interface, accidental fires and intentional fires are common, often reflecting cultural or religious practices (*48*). Just as there are many human ignition sources, there are many human factors that modify ignition probability, including suppression, fire management, and land-use change.

Although less frequent than human-caused fires in most of the world, fires from lightning can result in large burned areas, as lightning is the most common ignition source in remote, often unfragmented landscapes. A substantial proportion of fires in remote regions of Canada, the western United States and Australia are driven by lightning (*8*, *49*). Lightning incidence is projected to increase under climate change by more than 40% globally, with the greatest increases occurring in most forested areas of the planet (*50*).

### Landscape fire impacts

The framing of landscape fire impacts (along with the valuation or importance of those impacts) is a human construct (*51*). It is contingent on preferences, objectives, and perspectives; this makes assessing impacts deceptively challenging. Although there are many complexities involved, there are a range of methods and techniques for measuring and quantifying the impacts that occur at the fire front and beyond (*51*, *52*). Per-capita burned-area patterns (Fig. 2, bottom) are particularly informative for human impacts: Where burned area per person is high, suppression resources per capita are typically low, and exposure to transboundary smoke can dominate total health and economic impacts even when local burned area is modest. However, the overwhelming attention on the negative aspects of landscape fire impacts overlooks the essential roles that fire plays in many ecosystems and the broad range of cultural services that it provides or enhances (*53*).

A single fire can affect plant survival, germination, growth, and regeneration, hence vegetation community composition and structure. Similarly, fauna can suffer mortality during a fire, or after a fire due to lack of food or shelter resources, with recovery dependent on the reestablishment of food and shelter resources. Experimental burning programs have been used to study plant and animal regeneration [e.g., (*54*)], and field observations together with remotely sensed approaches have been used to look at how ranges of burn severity directly influence things such as soil microbiota (*55*), wildlife (*56*), and permafrost (*57*).

Going beyond the impacts of an individual fire are fire regimes, which describe the typical frequency, intensity, season, extent, and heterogeneity of fires over time in a given location [Fig. 3 and (*33*)] and which therefore influence medium- to longer-term vegetation community patterns. Plant functional traits that can help plants withstand fire (e.g., bark thickness) or regenerate after fire (e.g., resprouting, serotiny, and seed banks) have been studied in various vegetation types and can be a crucial consideration in modeling ecosystem impacts under climate change (*58*). High fire frequency can remove species that are unable to reach reproductive maturity before the next fire, whereas low fire frequency can lead to the loss of fire-dependent species (*55*). These changes can lead to shifts in flammability that reinforce directional changes in fire regimes; for example, short-interval fires may favor grass species, leading to a high frequency of lower-intensity fires (*59*). Vegetation changes are linked heavily with changes in fauna species and communities (*60*). Many ecological studies have used chronosequence approaches, medium-term prescribed burning experiments, or landscape-scale vegetation succession models to understand long-term fire patterns at the scale of the fire regime (*61*). Landscape succession models typically either simplify fire patterns [e.g., LANDIS (*62*)] or vegetation responses [e.g., FROST (*63*)].

Landscape fires have substantial direct impacts on humans and the built environment. Fatalities, large-scale structural losses, and losses of entire communities have been seen in many parts of the world, but systematic data collection remains limited (*19*, *64*). Studying and learning from situations where loss of life occurred (whether civilian or firefighter) is an important area of research (*65*). House destruction and damage, along with a wide variety of impacts on infrastructure (including transportation, communications, electricity, water treatment, hospitals, schools, and other public buildings), are a common occurrence, yet the ultimate economic and social consequences of those losses are far reaching and can dwarf the immediate impacts.

For structure loss, wind tunnels have been used to study ember ignition of roofing and other building components, experimental burns have measured how fire behavior affects structure, and postfire structural surveys are used in landscape fire–affected communities (*66*). Structure losses can inform empirically derived response functions to support house loss modeling (*67*, *68*). A variety of studies have looked at vegetation, housing arrangement, weather, cause of ignition, and risk mitigation actions as critical factors related to house loss (*69*, *70*). There have also been efforts to delineate what areas of a community or country are hot spots for fire impacts on humans—i.e., the wildland-urban interface (WUI); many studies have mapped the WUI globally or in various parts of the world (*71*). Some studies have also focused on recent trends in the fire exposure and/or extent of the WUI (*72*). Network modeling approaches have been used to investigate the various factors relevant to exposure to landscape fire in the WUI (*73*), and probabilistic modeling has been used to investigate fire likelihood in the WUI (*74*).

Indirectly, fire has impacts on many aspects of human health, social and cultural values, agriculture, industry, and the economy. Health impacts from smoke have been considered through analysis of hospital admissions or morbidity and mortality for respiratory and cardiovascular conditions (*75*). The mental health impacts of fire, smoke, and evacuations are beginning to receive attention (*76*), along with the extensive sociocultural impacts (*77*, *78*). Production through timber plantations and agriculture can be affected not only by loss of assets but also through the subsequent lag in production recovery. These impacts are salient to afforestation policies and related nature-based solutions (*79*). On the economic side, there are many impacts to consider, including industrial shutdowns, business closure, and fire management costs. Economic impacts of fires are difficult to calculate due to the many indirect costs and losses [e.g., tourism decline, real estate impacts, and water treatment costs; (*80*)] and secondary consequences of fire including the substantial personal costs of evacuations (*81*).

## CLIMATE CHANGE IMPACT ASSESSMENT

### Baselines

By definition, change involves a transition between two states. Understandably, the focus of climate change impact assessments is the second of these two states, i.e., the warmer and more extreme future that awaits us if we fail to make urgent and deep cuts to our greenhouse gas emissions (*1*). But this future is notable only insofar as it deviates from what has already occurred. Any understanding of projected climate change thus depends fundamentally on understanding the present and the past. For the purposes of climate change impact assessments, the present is often defined in terms of a recent climatological baseline (e.g., 1961 to 1990 and 1991 to 2020) or the industrial period (e.g., 1750 or 1800 onward). The “observational period” is often used as a baseline, but this measure is highly contingent and can range from many centuries [cf. cherry blossoms (*82*)], to many decades [temperature and precipitation (*83*)], and to just a few decades [satellite observations (*84*)]. Projected future climate is compared to present climate not just in terms of moments of the distribution (e.g., mean, median, and extreme values) but also variability (e.g., interannual). It is in the context of variability that paleoclimate studies have delivered powerful evidence about the nature of anthropogenic changes, such as in temperature and sea level rise (*1*). Evidence of substantial historical fluctuations in Earth’s climate underscores our climate system’s—and our—sensitivity to current and projected changes in greenhouse gas emissions (*1*).

### Earth system models

ESMs are the best current method to explore plausible climate futures. These models range considerably in terms of complexity and spatial resolution and in how well they capture the historical period (*85*). In addition to ESMs, considerable research has focused on downscaling global models to provide higher-resolution information for a given region, with grid resolutions of the order of tens of kilometers. The Intergovernmental Panel on Climate Change (IPCC) Sixth Assessment Report assesses some of these activities, many of which occur under the coordinated regional downscaling experiment (CORDEX). There are many downscaling activities independent of CORDEX that reach spatial resolutions of a few kilometers (*86*, *87*). Two common methods are dynamical downscaling, where regional climate models are forced at their boundaries by ESMs, and statistical downscaling, where fine-scaled data (e.g., from a weather station) is statistically linked to output at the scale of an ESM.

Dynamical downscaling adds value in regions of complex topography [e.g., (*88*)], but it necessarily imports errors from the global models and therefore may or may not provide generally more robust projections at higher spatial detail. Downscaling can also be undertaken using statistical approaches (*89*, *90*), but these approaches may not capture extremes well, particularly for precipitation. Moreover, in terms of future climate, methods that assume stationarity (i.e. that the relationships between fire and climate in the past and present will persist into the future) may provide a poor basis for future predictions because this assumption may not hold. Recent efforts to downscale ESMs using machine learning or artificial intelligence (ML/AI) show considerable promise (*91*, *92*). The potential of ML/AI is difficult to quantify, but one of the challenges in using ESMs and dynamically downscaled ESMs is small ensemble sizes, which ML/AI may help overcome. Results from ESMs and downscaling are often bias corrected with the assumption that the bias correction is stationary in a changed climate. However, while it is straightforward to interpolate information from ESMs to the kilometer scale, the spatial resolution that contains robust information is far more coarse, spatially variable, variable dependent, and very hard to quantify. In addition, ML/AI approaches tend to obscure insights into mechanisms and cause-effect relationships.

Irrespective of the approach used, using a single model, whether in the form of an ESM or a downscaled product, is generally an unreliable basis for decisions. Two approaches address this limitation: the creation of “ensembles of opportunity” where an experimental protocol is agreed and modeling groups choose to opt in as they wish [e.g., (*93*)] and the creation of large ensembles using a single ESM where one group defines an experimental protocol and runs a single model [e.g., (*94*)]. These two approaches are not interchangeable, and each has unique risks and benefits. Within the ensembles of opportunity, for instance, there are a wide variety of numbers of realizations used to explore internal variability. These provide useful information but are not commonly used in impact research, in part, because of discrepancies in study design that can bias results (e.g., aggregating data from 10 realizations from one model with 3 realizations from another model). Alternatively, developing large ensembles with a single ESM not only produces more internally consistent results but also inherently reflects any systematic biases associated with the selected ESM.

### Using climate model output

Climate models typically generate arrays of climate variables with one-time dimension and two or three spatial dimensions (e.g., time series of surface air temperature and precipitation or of air temperature at different atmospheric levels). For visualization and reporting purposes, spatial or temporal dimensions are often collapsed (e.g., by averaging over time or geographic area), although multiple models or emission scenarios may still need to be represented. Climate change projections are typically depicted as continuous time series (e.g., 1850 to 2100) or as a change from current conditions. The latter “delta” approach generally involves calculating statistics for reference historical periods (e.g., 1850 to 1900) and subtracting them from statistics based on reference future periods (e.g., 2081 to 2100) to arrive at the projected change value. An increasingly common variant of the delta approach involves reporting changes associated with benchmark levels of overall global warming such as 1.5°, 2°, or 4°C, regardless of when these benchmarks are projected to occur (*1*).

Postprocessing and analysis of climate model data vary in complexity, from simple spatial and temporal summaries of individual variables to multistep modeling chains that incorporate climate model output as part of a bigger process. At the simplest end are highly aggregated values common in IPCC assessment reports, such as maps of the projected change in annual, seasonal, or monthly temperature and rainfall and time series of the trajectory of the global mean of these variables through time. Descriptive statistics can also be used to investigate the potentially fine (i.e., subdaily) output available from climate models; often these include mean, median, minimum, maximum, and extreme values such as 95th percentile or days over (or below) threshold. A wide range of spatial aggregations of climate model outputs is possible, notwithstanding potential spatial resolution mismatches. Examples include continents, subcontinents (*95*), countries and administrative areas (*96*), biomes (*97*), ecosystem types (*98*), and land use or cover types (*99*, *100*).

Climate model output is frequently used to support impact assessments that extend beyond the narrow set of variables available from these models. In these cases, climate model outputs become inputs to other modeling processes. These downstream models range from simple to complex and from process-based to empirical, but all require a link between the climate system and the impact of interest. The simplest impact models produce climate indices, which can be calculated directly from climate model output and have been developed to represent many phenomena including modes of climate variability, climate extremes, biometeorology, aridity, and drought (*101*, *102*). More complicated models may require multiple additional inputs [e.g., crop yield models (*103*)] or have a computational expense similar to the climate models themselves [e.g., dynamic global vegetation models (*104*)]. Regardless of structure, impact models should be evaluated not just against observations but also in terms of their general robustness, which includes their sensitivity to climate and their ability to represent uncertainty (*105*). Even where climate change impact models have not been developed, any link between the climate and some other system can serve, at least implicitly, as a basis for assessing potential impacts of climate change.

### Thorny issues in climate change impact assessment

The climate change impact assessment process is beset by uncertainty and complexity, sometimes irreducibly so. Transparency about limitations is essential for developers and users of these assessments. Here, we present some key issues for developers and users of general climate change impact assessments.

#### Model evaluation

There is no consensus on what makes a “good” or “bad” model despite major assessment processes led by the IPCC (*85*, *106*). A major challenge for users of ESMs is the rapid increase in the variety of models. In 1990, when the first IPCC report was published, three coupled climate models existed—now, there are 61 (*107*). While more models might seem beneficial, these models are not fully independent, potentially biasing results when multiple seemingly independent models are averaged or otherwise combined. Despite this, there are no standardized methods or best practices for bias correction and different methods can lead to very different outcomes (*108*).

#### Using multiple models

There is no consensus about ensemble design, which involves decisions about emission scenarios and model selection criteria including model performance, model independence, and the range of possible climate futures considered (*109*). Users of climate projections may be unaware of how a particular ensemble was designed, limiting their ability to draw reasonable inferences from it. Where climate change model output forms part of a long or complex chain of models, there is the risk of multiplication of uncertainty and errors, including through mismatches in the spatial resolution of different data layers.

#### Application

Despite the limits of ESMs, scenarios, downscaling, and impact assessment modeling chains, there is strong demand for—and a growing supply of—products that aim and/or claim to fill these knowledge gaps. However, it is not currently feasible to develop highly granular risk– or outcome-based estimates of future fire. There are options that can be developed to explore plausible futures, including sensitivity testing, stress testing, and storylines [plausible, physically self-consistent scenarios intended to be illustrative rather than representative (*110*, *111*)]. Despite this, developers and users should be aware that selecting and communicating individual futures from within a fundamentally uncertain set of plausible futures are deeply challenging.

## ASSESSING CLIMATE CHANGE IMPACTS ON LANDSCAPE FIRE

### Modeling future fire

**Fig. 4.**
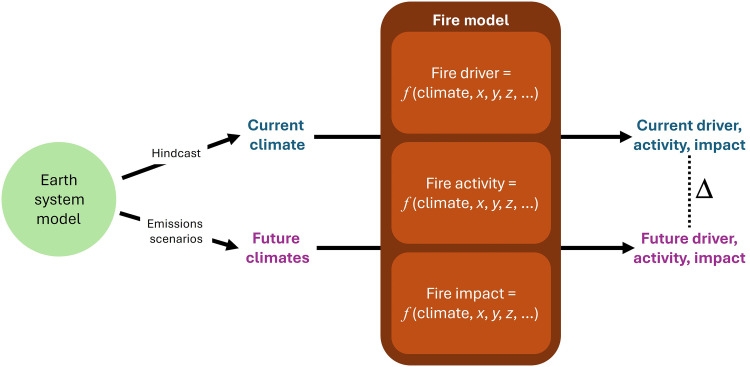
Visualization of climate change impact assessment for landscape fire. Assessment combines fire models (brown box) with ESMs (green circle). Fire models can take a range of forms but must include a link between the climate system and some aspect of fire. ESMs can be used to generate plausible future climates based on specific emission scenarios. This future climate information can then be used to “drive” the fire model, resulting in projections of future fire. The difference (delta) between modeled present and future conditions is often used as a measure of the degree of any projected changes. The difference between modeled and observed fire (or climate) is referred to as bias and is not shown here. ESMs increasingly include models of fire activity.

To use ESMs to explore plausible fire futures, either fire must be explicitly represented in the model or a relationship must be established between the climate/weather system and landscape fire activity (Fig. 4). Simulating fire within an ESM is challenging, and intercomparisons between models point to large variations between burned area projections (*112*). Many of the process-based fire models have been improved in the past decade, partly thanks to the Fire Model Intercomparison Project (*113*, *114*). In the next IPCC round, for the first time more than half of the contributing climate models will have an interactive fire module and can explore climate-fire relations and feedbacks. Alternatively, using the relationship approach, ESM-derived changes in climate/weather can be used to predict corresponding changes in fire. Empirical models are regularly developed for this purpose (*115*, *116*). Of perhaps greater interest are process-based models that have a wide user community and aim to represent a range of relevant factors, including fire growth models, landscape fire models, coupled atmosphere-fire models, and most recently ML models.

Fire growth models (also known as fire behavior models or fire simulators) use relationships between weather, fuel spatial distribution, type, load, moisture, and topography to estimate fire behavior variables such as rate of spread and intensity. These models are often used by fire management agencies for tactical and strategic applications for an individual fire. For example, FARSITE (*117*) has been used for operational and research purposes for decades. Fire growth models have been used to study the impacts of climate change on fire activity in various regions with findings ranging from a modest increase up to an eightfold increase in area burned by the end of the 21st century (*118*, *119*). Landscape fire models can simulate multiple fires over the landscape and can be one of the most effective tools for studying the relationships between climate/weather, fire, and vegetation. The LANDIS model *et al.* (*62*) has been widely used to study fire and windthrow and harvest disturbance regimes on landscape pattern and structure. These landscape fire models have also been used to explore the potential impacts of climate change on fire activity and vegetation (*59*). There are hundreds of fire growth and landscape fire models that have been developed for many applications so care should be taken to ensure that the right model is used for an appropriate application.

In contrast with fire growth and landscape fire models, coupled fire-atmospheric models use physical or empirical modeling of fire processes in the atmospheric boundary layer with a computational fluid dynamics model of the atmosphere. They are used to improve our understanding of key physical (and to a lesser extent social) processes driving fire dynamics and potentially enable better predictability of fire behavior across a wide range of conditions (*120*). For example, FIRETEC (*121*) models the coupling between fire, atmospheric conditions, and topography. These coupled models allow fire itself to influence local weather (e.g., wind speed and direction) that then alters fire behavior through feedback processes. The addition of such feedbacks may be essential for accurately modeling landscape fire spread, especially for extreme fire behavior events.

Last, a wide range of ML/AI methods have been applied to predict fire growth including Artificial Neural Networks, Bayesian Networks, Genetic Algorithms, K-Nearest Neighbor, and Random Forests. The ML/AI approach to fire modeling is growing rapidly and includes modeling of fuels, fire detection and mapping, fire weather and climate change, fire probability, fire behavior prediction, fire effects, and fire management (*122*, *123*). Several studies have used ML approaches to estimate the impact of climate change on fire activity or at least fire weather. For example, Moritz *et al.* (*124*) used MaxEnt to project future fire probability globally. Despite the ability of ML methods to learn from data without explicit causal or process modeling, expertise in landscape fire science is necessary to ensure realistic representations of fire processes across multiple scales. Moreover, the complexity of some ML methods, such as deep learning, requires an extensive and sophisticated knowledge of their application.

### Modeling the future of fire’s drivers

Given fire’s complexity, a common approach to climate change impact assessment is to project changes to just one of the key drivers of landscape fire (fuel amount, fuel dryness, weather, and ignition), effectively holding the other drivers constant (Fig. 4). Weather and climate are central to landscape fire, and ESMs are central to climate change impact assessment. As a result, there has been an abundance of studies examining changes in fire weather in the context of projected changes in climate, often using fire danger rating systems (*14*). Broadly speaking, projections indicate a substantial increase in the probability of weather and climate conditions that promote extreme fire activity, e.g., up to a 10-fold increase in Europe (*125*). Climate change is recognized to have several impacts on atmospheric instability, primarily through enhanced surface heating and increased atmospheric moisture. These changes can increase the energy available for convection, potentially intensifying storms. In addition, alterations in large-scale circulation patterns (e.g., weakening of the jet stream) may shift the distribution of instability and wind patterns, potentially leading to more extreme weather and fire events (*126*). One notable impact already observed is an almost doubling of clear-air turbulence due to climate change (*127*). Clear-air turbulence, which substantially affects aviation, is caused by vertical wind shear events when two air masses overlap and move at different speeds or in different directions and are the results of a generally more unstable atmosphere. We could, therefore, hypothesize that an increase in the probability of pyrocumulus formation is plausible. Emerging evidence suggests that there may be a link between pyrocumulus occurrences and climate change, but more work in this area is needed (*128*).

Atmospheric demand for water can be readily computed from ESMs and, as noted above, is closely linked to fuel moisture (*129*). Despite this, there are relatively few studies that focus explicitly on future fuel moisture [e.g., (*130*, *131*)], with many instead incorporating fuel moisture into broader predictions of fire danger or activity. Simplifying climate change assessments by focusing on a single driver is problematic in the case of fuel, as one complexity (fire) is traded for another, arguably far greater one, i.e., vegetation. Nevertheless, there are some indications of how vegetation and fuel may change with continued climate warming. In some areas, the expansion of flammable vegetation into regions where it was previously sparse (e.g., tundra to boreal transition zones) could lead to an increase in fire activity (*21*). Conversely, the loss of forest cover in regions experiencing extreme drought or heat stress may reduce fuel availability, potentially decreasing fire activity while increasing susceptibility to erosion and land degradation (*132*). Empirical and process-based models have been used to predict future fuel (*133*), with dynamic global vegetation models representing a special case.

With respect to ignitions, projecting future human-caused ignitions is challenging. Large changes in human behavior are likely in a changing climate, but these aspects are often excluded from climate analyses due to the intrinsic difficulties of including nonphysical processes. There are very few studies examining how social behavior has shifted and may continue to shift, as well as the potential modifications these changes could bring to current fire regimes. Nevertheless, because fire weather is a strong predictor for a range of anthropogenic ignition causes (*134*), continued warming is likely to increase these ignitions, all other things being equal. ESMs have been used to investigate how lightning frequency will change as the planet warms. Lightning frequency was found to increase by up to 30% per degree of global warming over intact extratropical forests, suggesting that climate change increases the risk of landscape fire ignitions (*135*, *136*). Moreover, especially in the Arctic regions, this will be in addition to a projected increase in ignition efficiency by 9% in Alaska and 28% in the Northwest Territories of Canada per °C of warming by the end of the century (*135*). This means a growing likelihood of ignitions in these areas.

### Modeling future fire impacts

The key challenge for projecting the impacts of fire under climate change is the addition of potentially complex, multistep chains of causality to the already complex phenomenon of landscape fire (Fig. 4). In general, the further removed from the direct effects of fire, the harder it is to project impacts. Compared to fire activity and the key drivers of fire, there has been relatively little study of future fire impacts, although this is likely to change rapidly in the coming years.

Under climate change, the costs of landscape fire and fire management are likely to increase markedly. For example, one study found that fire suppression costs in those rare, but very busy fire seasons will simply be a common, almost annual occurrence (*137*). Another study found that average annual costs of treatments and impacts will substantially increase under future climates (*138*). Empirical relationships between fire, smoke, and human health have been linked with risk models (*139*), finding that smoke health costs of fire are likely to increase. The same kinds of risk models have been used in southeastern Australia to explore future risks to obligate-seeder forests, which are vulnerable to increased fire frequency (*63*).

It is easier to project impacts when they are common and direct, and there is strong empirical evidence that can be used to model or forecast future fire risk to values. However, where the impacts are indirect and influenced by social, environmental, economic, and cultural factors, the relationships are harder to study due to the overriding effects of local conditions. Last, where there are feedbacks from fire regimes to the values, these become more challenging to measure empirically and model into the future.

### Thorny issues in projecting climate change impacts on landscape fire

The relationship between landscape fire and climate change is multifaceted, multiscale, dynamic, and full of feedbacks. This complexity and uncertainty make modeling future fire a challenging task. Here, we present some key issues for developers and users of climate change impact assessments for fire.

#### Baselines

**Fig. 5.**
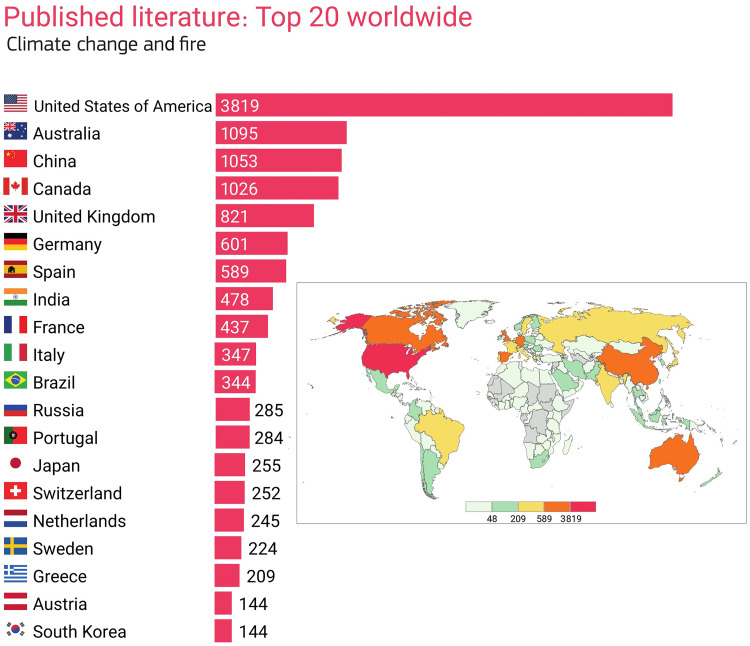
Top 20 countries with the most journal articles published on the topic of climate change and landscape fire. See the Supplementary Materials for details of search term.

Climate change impacts depend on properties of the prevailing fire regime, including the relative importance of fuel, dryness, weather, and ignition; the interplay between these factors and how they can constrain fire is complex and varies spatially (*32*, *140*). This limits our understanding of current baselines and thus our ability to interpret potential changes. Furthermore, much of our knowledge of fire and its interactions with the weather/climate system is biased toward studies whose authors were based in North America, Australia, and Western Europe (Fig. 5). These blind spots can be locally, regionally, and even globally important (cf. the tropics).

#### Nonstationarity

Nonstationarity means that fundamental processes can change over time in ways that may not be fully understood. This is an issue for any model that aims to predict the future based on fire-weather/climate relationships in the present or past. These predictions assume that observed relationships will persist in the future, a hypothesis that may be false and is not easily testable. Process-based models may have an increased capacity to deal with stationarity compared to simple empirical and highly complex physical models, but more research is required (*141*). A related point is the possibility of new fire or climate/weather conditions for which there are no historical analogs (*142*). In either case, predictions may become unreliable or more uncertain.

#### Working with ESMs

ESMs use spatial resolutions of around 100 × 100 km, which makes them of limited value to examining fire risk. These models tend to overestimate atmospheric water vapor (thereby underestimating fuel dryness) in some regions (*143*); underestimating this key driver of fire activity may mean impacts will ultimately be higher than predicted. Variables that depend on large-scale synoptic scale systems (e.g., heatwaves) might be well projected but other fire-relevant phenomena remain poorly resolved [e.g., fronts, sea breezes, slope and valley winds, and wind gusts (*144*)]. In addition to this, there are no “representative conflagration pathways,” i.e., agreed scenarios for fire (analogous to emissions scenarios) that include human interventions such as prescribed burning and that can be used to guide impact assessment [although see (*145*)]. As noted above (see Climate change impact assessment section), choice of model and scenario directly affects projections of future fire activity. It may be helpful for those using ESM output to understand where their selected model(s) sit within the overall change space [e.g., (*146*)].

Downscaling and bias correction can add value to ESMs, but there are limits. There is little guidance as to which ESMs or downscaling products are “best” to use in the case of landscape fire. Bias correction for landscape fire analysis is challenging because it may draw on multiple, potentially codependent variables with considerably different observational records (cf. temperature, rainfall, humidity, and wind speed, common ingredients of fire danger rating systems). Potential responses include using multiple models (not as an average but individually), evaluating models for the specific purpose, testing for model independence, aggregating data across models as distinct from averaging, and using specialized methods (*147*). Bias correction may not even be possible for important fire-relevant variables as fuel loads, fuel dryness, and even wind due to a lack of long-term observations. ML/AI models may potentially provide better fits to data than traditional statistical approaches, especially where they can draw on large datasets, e.g., ESMs and satellites (*148*).

#### Understanding and managing impacts

Changing fire environments may push risk management into uncharted territory, raising the urgency of understanding impacts. Per-capita perspectives also matter operationally: Remote, sparsely populated fire landscapes—which dominate the per-capita map (Fig. 2, bottom)—are precisely where suppression capacity saturates first under concurrent events, making escalation with warming harder to contain and increasing the probability of multiregional smoke events. Moreover, new areas of the world are emerging as fire prone (*149*), and there are more overnight burning events (*150*), overwintering fires (*151*), and longer and more extreme fire seasons (*19*). Projections do not consider unknown unknowns, i.e., totally unexpected surprises or sudden changes in fire activity.

Our methods for mitigating risk at tactical and strategic levels are also likely to change over time, which could substantially affect the predicted effects of climate change. Global urbanization will increase the demands to protect communities (*72*), fire management policy changes can affect fire activity (*152*), and the consequences of climate change will lead to fire activity that exceeds the limits of suppression resource capacity (*119*, *131*, *153*). These pressures are forcing a fundamental rethink of how we manage fire.

#### Feedbacks

Because of the influence of past fire on future fire, potential increases (decreases) in fire may be partially compensated by decreases (increases) elsewhere, but this requires testing and is complicated by the influence of various bottom-up and top-down drivers (*154*, *155*). At the scale of the global climate system, it is not yet clear whether landscape fires will have a positive or negative feedback to global warming. Feedbacks can stem from deep burning tropical and boreal peatland fires and their substantial emissions (*156*), smoke and greenhouse gas emissions from fire, black carbon aerosol and deposition, immediate and long term impacts of fire on permafrost and the release of hydrates, changes to lightning occurrence, albedo, and the effects of stratospheric smoke and moisture from increased convective energy and pyrocumulonimbus events (*157*).

#### Interdisciplinarity

The multifaceted nature of landscape fire and its impacts means that a full understanding of the processes of interest requires expertise in multiple disciplines. We have already mentioned a range of topics (e.g., atmospheric physics, botany, ecology, economics, fire behavior modeling, health and medical sciences, meteorology, paleo science, and remote sensing), but others are important including pests, disease, and invasive species. The direct and indirect influence of humans over fire and its impacts loom large. Key examples include fire suppression, prescribed and cultural burning, land-use changes, forest clearing and broader policy, and legal and governance frameworks. These human dimensions of fire are not easily incorporated into models (*158*), although exceptions include scenarios of land-use change (*159*) and population growth (*160*).

Integrating these insights requires collaboration and communication across traditional academic and administrative boundaries, sectors, and knowledge systems to understand differences in methods, norms, data quality, standards, and so on. This collaboration requires additional commitments of time, effort, and funding and is often actively disincentivized. From a practical standpoint, there is also no agreement on how best to combine results from different methodological approaches. Methodological diversity can be an asset, but it requires additional consideration, deliberation, and potentially innovation.

## TOWARD EFFECTIVE CLIMATE CHANGE IMPACT ASSESSMENTS FOR LANDSCAPE FIRE

Assessing the impacts of climate change on landscape fire incorporates methods from climate modeling and landscape fire modeling (Fig. 4). It therefore requires an understanding of principles, methods, limitations, and knowledge gaps in these two fields. Both fields are intrinsically multidisciplinary, which argues for diverse, multidisciplinary teams to carry out this work. Without an understanding of landscape fire, practitioners and their audiences are vulnerable to a range of avoidable problems, such as not addressing feedbacks or ignoring important nonclimate drivers of landscape fire risk (see Thorny issues in climate change impact assessment section). Undertaking this work without understanding how climate change impacts are assessed leaves the same groups vulnerable to a different set of issues, including inadequate consideration of the range of possible future changes in climate and false confidence about the precision or spatial resolution of impacts (see Modeling future fire impacts section). To make these issues more tangible, we provide two examples of potential research that could contribute to a climate change impact assessment for fire, both in the form of storylines (Box 1). Each touches on only a fraction of the issues rose here, but in keeping with the spirit of storylines, they are self-consistent examples of efforts to engage with current knowledge and methods in landscape fire and climate change modeling.

Box 1. **Two storyline approaches to climate change impact assessment for landscape fire.**

Storyline 1: Probability of a Black Summer–type event under current and future climate change

**Fig. 6.**
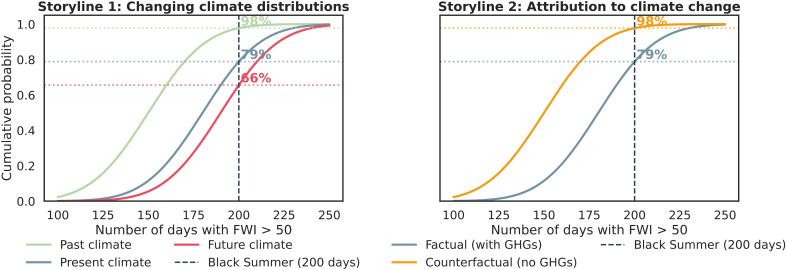
Hypothetical data illustrating the two storyline-based approaches to climate change impact assessment for landscape fire. Both examples use the FWI to represent fire weather conditions and an index threshold value of 50 to represent extreme conditions. The study area and time is the Black Summer fires of 2019 and 2020 in eastern Australia. Storyline 1 (left) shows the historical context of a Black Summer–type event. In the past climate example (e.g., 1950 to 1980; green line), an event with 200 days above the FWI threshold would have fallen at the 98th percentile. In the present climate (e.g., 1990 to 2020; blue line), the same 200-day event lies at approximately the 79th percentile, while in a future climate scenario (e.g., 2080 to 2100; red line), an FWI of 50 represents the 66th percentile, reflecting a projected increase in extreme fire weather conditions. Storyline 2 (right) shows the attribution of increased risk using ESM simulations. In the factual world with human-induced greenhouse gasses (GHG; blue line), the 200-day Black Summer event falls at approximately the 98th percentile, whereas in the counterfactual world without human influence (yellow line), the same event would have been at the 79th percentile, indicating that such extreme fire conditions would have been much rarer without anthropogenic climate change.

The study topic considers whether climate change is increasing the likelihood and severity of extreme fire conditions. The specific question is will the weather conditions associated with the 2019–2020 Black Summer fires in eastern Australia represent an outlier relative to the historical record and whether those conditions will become more likely under future climate change. A key consideration is how to characterize the extremity of the Black Summer conditions. One approach is to use established fire danger metrics such as the Forest Fire Danger Index (FFDI) or the Fire Weather Index (FWI). These metrics could be sourced from regional or global reanalysis datasets or from long-term station observations. Several complementary methods are available for defining how extreme conditions were. A fixed threshold (e.g., an FWI or FFDI value of 50) could be used to examine whether the number of days above this threshold was exceptional during the Black Summer. In contrast to this somewhat arbitrary threshold, statistical approaches such as percentiles or sigmas could also be used. Percentiles allow identification of days when index values fall in the top 1% of the historical distribution. Sigmas measure how many SDs (σ) an event lies from the long-term mean, e.g., a 5-sigma event represents truly exceptional fire danger. The choice of reference period is crucial, as it contextualizes events in the relevant climatology. A standard baseline for present-day climate, such as 1990 to 2020, provides a distribution against which the rarity of an event can be assessed. Examining previous periods, for example, 1950 to 1980, helps understanding of how the odds of such an event have changed over time. Using future climate simulations further allows us to estimate the probability of observing a similar extreme event under projected conditions (Fig. 6).

Storyline 2: Attribution of a Black Summer–type event to anthropogenic forcings

The study topic is the extent to which observed increases in the likelihood and severity of extreme conditions can be attributed to anthropogenic forcings and emissions. The specific question is whether, and if so by how much, human-caused greenhouse gas emissions have changed the likelihood of weather conditions associated with the 2019–2020 Black Summer fires in eastern Australia. Attribution analysis compares the observed or reanalysis-based distribution of extreme fire weather indices with distributions generated by climate models under different forcing scenarios, for example, using Overview of the Coupled Model Intercomparison Project Phase 6 simulations. In this method, we compare the actual world, which includes anthropogenic greenhouse gas emissions and other human influences, with a counterfactual world where human influences are removed, representing a preindustrial climate. Using the threshold established in storyline 1, we could test whether the likelihood of exceeding it has changed between these two worlds. For instance, if in the counterfactual world only about 1% of days exceed the threshold, but in the actual world 5 or 10% of days exceed it, then the odds of experiencing Black Summer–type fire weather could be said to be higher due to human-induced climate change. This approach would highlight the extent to which anthropogenic forcing has shifted the entire distribution of fire danger toward higher values, making days that used to be exceptionally rare (e.g., top 1%) occur much more frequently in today’s climate (Fig. 6).

A more fundamental limitation is that scientific research plays a necessary but insufficient role for supporting knowledge, decisions, and actions outside academia (*161*). This is most obvious in the case of climate change, where decades of science have been—and continue to be—met with a wall of resistance from powerful interests and entrenched ideologies (*162*). By now, it is abundantly clear that simply assessing the impacts of climate change in a particular area does not necessarily lead to concrete, meaningful steps to mitigate or adapt to these impacts. There is no equivalent of a fossil fuel lobby in landscape fire, but there are multiple, sometimes incompatible alternative framings of the landscape fire problem, including whether it is even a problem at all (*53*, *163*). Not all of these framings are equally privileged by maintaining the status quo, which, for example, tends to favor expenditures on suppression over recovery and risk reduction (*152*) and to marginalize Indigenous fire knowledge (*25*, *164*). First Nations approaches to fire may not sit easily within a risk management paradigm (*139*), and more work is needed to include, support, and value this knowledge in its own right (*165*, *166*). Communicating scientific knowledge about landscape fire and translating it into meaningful action are challenging, especially given the diverse set of stakeholders that influence and are affected by landscape fire. This situation argues not just for multidisciplinary teams but also teams that span different sectors and knowledge systems, including Indigenous fire knowledge. In the coming years, building upon existing knowledge will be crucial for developing robust climate change impact assessments for landscape fire, but the most influential outcomes from this work may come through innovation on translating this knowledge into effective decisions.
