# Supplementary material for: Gazing into the flames: A guide to assessing the impacts of climate change on landscape fire
Source: Sci Adv. 2025 Dec 19;11(51):eadz2429. doi: 10.1126/sciadv.adz2429 (PMC12716398; doi:10.1126/sciadv.adz2429)
Supplement: Supplementary file 1 — Legends for data S1 and S2 [file sciadv.adz2429_sm.pdf]

Science Advances
AAAS

Supplementary Materials for

Gazing into the flames: A guide to assessing the impacts of climate change on landscape fire

Hamish Clarke *et al.*

Corresponding author: Hamish Clarke, hamish.clarke@unimelb.edu.au

Sci. Adv. **11**, eadz2429 (2025)
DOI: 10.1126/sciadv.adz2429

The PDF file includes:

Legends for data S1 and S2

Other Supplementary Material for this manuscript includes the following:

Data S1 and S2

Data S1. (separate file)

Data used to construct Figure 1. Data is a CSV file returned from a Scopus keyword search of journal articles published on the topic of climate change and landscape fire (Scopus, April 3 2025). Search term was (TITLE-ABS-KEY ("climate change" OR "global warming") AND (TITLE-ABS-KEY (“wildfire “ OR “bushfire “ OR "landscape fire" OR "forest fire" OR "grass fire" OR "wildland fire" OR "biomass burning")). Filter is articles per year.

Data S2. (separate file)

Data used to construct Figure 5. Data is a CSV file returned from a Scopus keyword search of journal articles published on the topic of climate change and landscape fire (Scopus, April 3 2025). Search term was (TITLE-ABS-KEY ("climate change" OR "global warming") AND (TITLE-ABS-KEY (“wildfire “ OR “bushfire “ OR "landscape fire" OR "forest fire" OR "grass fire" OR "wildland fire" OR "biomass burning")). Filter is articles by country.
